# Occurrence and expression of novel methyl-coenzyme M reductase gene (*mcrA*) variants in hot spring sediments

**DOI:** 10.1038/s41598-017-07354-x

**Published:** 2017-08-03

**Authors:** Luke J. McKay, Roland Hatzenpichler, William P. Inskeep, Matthew W. Fields

**Affiliations:** 1 Center for Biofilm Engineering, Montana State University, Bozeman, MT 59717 USA; 2 Department of Land Resources and Environmental Sciences, Montana State University, Bozeman, MT 59717 USA; 3 Department of Chemistry and Biochemistry, Montana State University, Bozeman, MT 59717 USA; 4 Department of Microbiology and Immunology, Montana State University, Bozeman, MT 59717 USA

## Abstract

Recent discoveries have shown that the marker gene for anaerobic methane cycling (*mcrA*) is more widespread in the *Archaea* than previously thought. However, it remains unclear whether novel *mcrA* genes associated with the Bathyarchaeota and Verstraetearchaeota are distributed across diverse environments. We examined two geochemically divergent but putatively methanogenic regions of Yellowstone National Park to investigate whether deeply-rooted archaea possess and express novel *mcrA* genes *in situ*. Small-subunit (SSU) rRNA gene analyses indicated that Bathyarchaeota were predominant in seven of ten sediment layers, while the Verstraetearchaeota and Euryarchaeota occurred in lower relative abundance. Targeted amplification of novel *mcrA* genes suggested that diverse taxa contribute to alkane cycling in geothermal environments. Two deeply-branching *mcrA* clades related to Bathyarchaeota were identified, while highly abundant verstraetearchaeotal *mcrA* sequences were also recovered. In addition, detection of SSU rRNA and *mcrA* transcripts from one hot spring suggested that predominant Bathyarchaeota were also active, and that methane cycling genes are expressed by the Euryarchaeota, Verstraetearchaeota, and an unknown lineage basal to the Bathyarchaeota. These findings greatly expand the diversity of the key marker gene for anaerobic alkane cycling and outline the need for greater understanding of the functional capacity and phylogenetic affiliation of novel *mcrA* variants.

## Introduction

Archaea are the primary drivers of CH_4_ cycling on our planet. Each year, methanogens produce approximately 1 Gt of CH_4_, of which 80% is subsequently oxidized anaerobically by methanotrophic archaea in the same habitats^1^. Because of its importance as an intermediate in the biological carbon pump^2, 3^ and its role as a potent greenhouse gas, it is crucial to understand microbial sources and sinks of CH_4_. The discovery of near-complete methanogenesis pathways in the genomes from the recently described Bathyarchaeota^4^ and Verstraetearchaeota^5^ has drawn into question our view, held for over four decades, that the ability to generate or oxidize CH_4_ anaerobically is limited to a single archaeal phylum, the Euryarchaeota. Both Bathyarchaeota and Verstraetearchaeota lack cultured representatives, but are widespread across diverse environments^5–7^. Moreover, recent analysis of *Candidatus* Syntrophoarchaeum implicates the involvement of the *mcr* complex in butane metabolism^8^, underscoring the need for further investigations into coenzyme M reductase functions and archaeal carbon cycling.

Although geothermal systems are hypothesized as life’s first habitat/s^9^ and methanogenesis is thought to be an ancient metabolism^10, 11^, very little evidence of methanogenesis has been reported in Yellowstone National Park (YNP, Wyoming, USA), the planet’s largest and most diverse terrestrial geothermal ecosystem. While investigations of methane cycling in other continental geothermal ecosystems have recovered *mcrA* genes and enrichments of methanogens^12, 13^, only one methanogen, *Methanothermobacter thermoautotrophicus*, has been isolated from the YNP ecosystem^14^. Further, metagenome studies in YNP have not yet identified habitats containing diverse *mcrA* (α-subunit of methyl coenzyme M reductase) genes^15^. Here, we investigated the possibility that CH_4_ cycling may be a more dominant feature in thermal sediment communities of YNP than previously thought. We examined two chemically and hydrothermally distinct regions of YNP where methanogenesis has previously been inferred, either by successful isolation at Washburn Hot Springs (WS)^14^ or by recovery of taxonomic marker genes of methanogens in the Heart Lake Geyser Basin (HL)^16^. We characterized the archaeal communities present in several selected sites by determining the occurrence of SSU rRNA genes and transcripts. Noting that CH_4_ cycling organisms are commonly observed in subsurface environments, we investigated surface as well as subsurface thermal sediments for euryarchaeotal- and bathyarchaeotal-like *mcrA* genes using newly developed PCR primers that were designed to amplify a greater diversity of known *mcrA* sequences based on recently reported metagenomes^4^.

## Results and Discussion

### Physicochemical attributes of thermal features

Two geographically and geochemically distinct thermally active regions of YNP (Washburn Hot Springs (WS) near Mt. Washburn and Heart Lake Geyser Basin (HL) at the base of Mt. Sheridan) were evaluated for archaeal community structure and the occurrence and expression of novel *mcr*A sequences. WS and HL lie along the margin of the most recent (0.6 Ma) caldera, with WS on the north-eastern border and HL on the south-central border^17^. WS is a vapor-dominated acid-sulfate system where subsurface carbonate-rich steam heats and reacts with spring fluid to yield anoxic pools replete with sodium bicarbonate, sulfide and ammonium. The high concentration of NH_4_^+^ at WS is due to hydrothermal fluid contact with marine sediments during migration and is among the highest measured in YNP^18, 19^. Discharge pools at WS span a temperature range of 64–91 °C, and the major gases of the two thermal pools sampled include H_2_, CH_4_, and up to 94% CO_2_ (Table 1). By contrast, HL contains high-temperature, water-dominated systems that discharge alkaline fluids^17^; the three hot springs sampled at HL span a pH range of ~ 8.2–8.6 and a temperature range of 34–73 °C. Average CH_4_ concentrations at HL and WS are significantly different from one another based on one-tailed equal and unequal variance t-tests (p = 1.95 × 10^−10^ and p = 2.17 × 10^−6^, respectively), but they fall within the same order of magnitude of between 1 and 6% of source gas concentrations and are thus proportionally similar. Oxygen availability can be inferred from measurements of dissolved sulfide (DS), which indicate that HL and WS are geochemically divaricate: DS values reported previously for the springs sampled at HL were below detection^16^ (supplementary text) whereas values from WS0 range from 160 μM^15^ to 231 μM DS (Supplementary Table S3). Gas discharge at WS contains 85–94% CO_2_, which contrasts with HL where lower levels of CO_2_ (Table 1) are likely replaced by N_2_ (not measured). Elevated CO_2_ at WS contributes to carbonate-buffering and a lower pH range of 5.8–7.0 as compared to the alkaline (pH 8.2–8.6) springs of HL. Previous^15, 16^ as well as the present geochemical measurements from WS and HL detected the necessary substrates for hydrogenotrophic methanogenesis, which is consistent with the isolation of the CO_2_ reducing methanogen, *M. thermautotrophicus* from WS^14^. These observations together with the previous detection of sequences related to hydrogenotrophic, acetoclastic, and methylotrophic methanogens at HL^16^ indicate that both geothermal systems are appropriate sites for a targeted investigation of archaea with metabolic potential for CH_4_ cycling.

**Table 1:** Physicochemical attributes of Washburn (WS) and Heart Lake Geyser Basin (HL) hot springs.

Site	Temperature (°C)	Source Gases (%)			
		pH	CO_2_	H_2_	CH_4_
WS0	64	6.5	93.81 ± 2.93	2.42 ± 0.09	6.00 ± 0.19
WS1	77	5.8	85.21 ± 5.40	2.94 ± 0.05	4.82 ± 0.08
WS3	91	7.0	nd	nd	nd
HL9	61	8.6	3.76 ± 0.19	0.01 ± 0.00	1.18 ± 0.02
HL10	34	8.2	31.66 ± nd	0.02 ± nd	0.75 ± nd
HL11	73	8.5	nd	nd	nd

Temperatures and pH values are listed for each hot spring sampled at WS and HL. Geothermal source gas bubbles were captured and analyzed for the presence of CO_2_, H_2_, and CH_4_ (±values are equal to the standard deviation of the mean percentage value based on quadruplicate resampling). Time and weather constraints prevented replicate sampling from being collected at HL10. Collection of source gases was not possible at WS3, which is not fully submerged in spring fluid, and HL11, which did not produce observable bubbles (nd = not determined).

### SSU rRNA gene survey of Archaea

**Figure 1:**
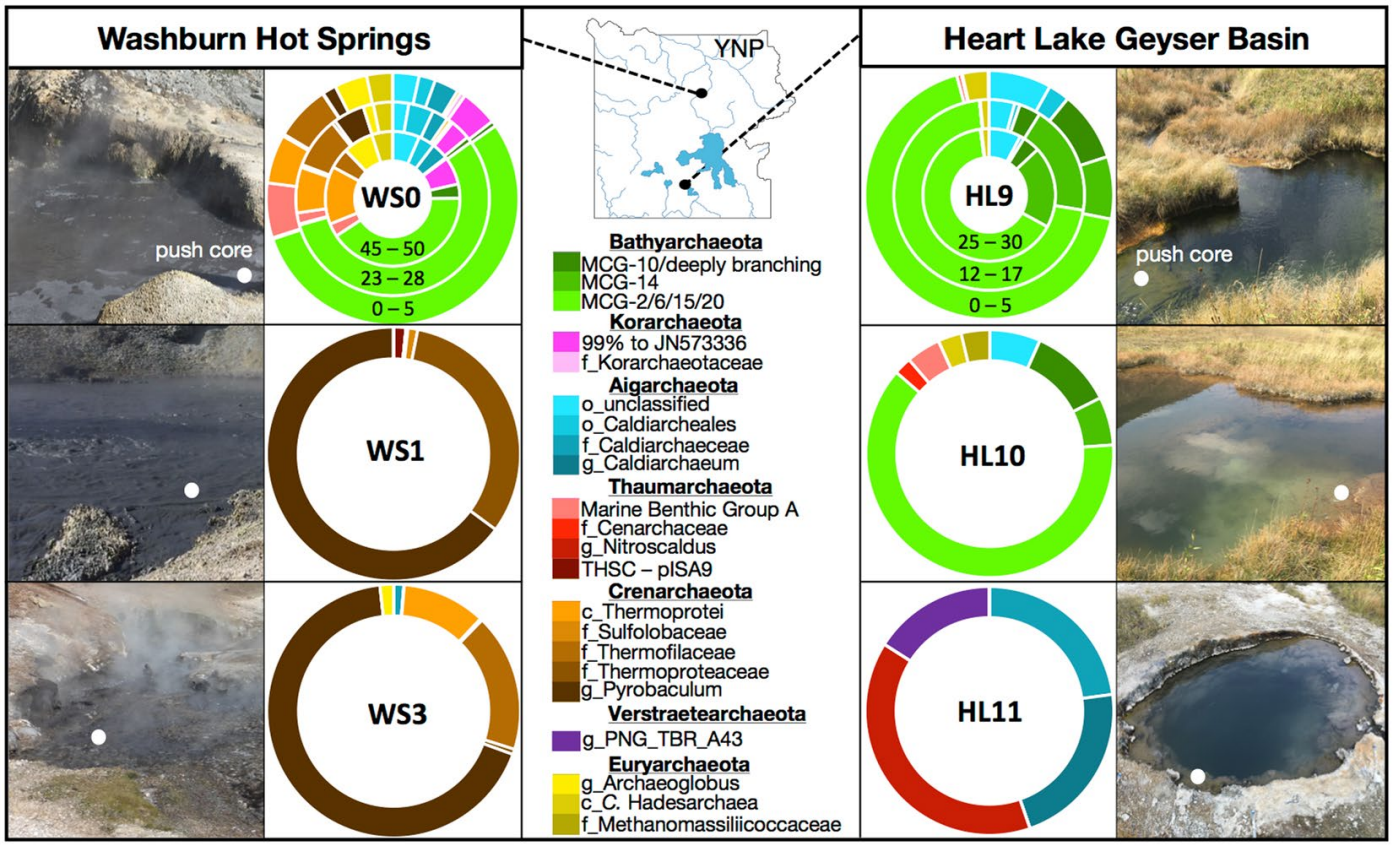
16S rRNA gene diversity of Archaea in physicochemically and regionally distinct Yellowstone hot spring sediments. Taxonomic groupings of archaeal OTUs representing >0.1% of the sequences recovered from Washburn Hot Springs (WS) and Heart Lake Geyser Basin (HL). At one spring from each region—WS0 and HL9—a sediment push core was sectioned in three layers for separate microbial analysis, and the sediment depth in cm is labeled on each respective section of the circle chart with the deepest section closest to the center. Photographs of each hot spring are adjacent to diversity charts, and white circles indicate the location of sampling. Taxonomy is grouped by phylum and the next highest resolution taxonomic group possible is listed beneath the phylum as a separate color (c = class; o = order; f = family; g = genus). For Bathyarchaeota, MCG subgroups are listed according to classifications by Kubo and Lloyd *et al*. (2012) and Lazar *et al*. (2015), except for MCG-20, which is proposed in this study (Supplementary Figure S1). The map in this figure was obtained from the public domain national park maps available from the US National Park Service (https://www.nps.gov/hfc/carto/PDF/YELLmap2.pdf; licensing: https://www.nps.gov/hfc/carto/data-sources.cfm) and simplified with Adobe Illustrator CC 2015 (Adobe Systems; www.adobe.com/illustrator).

DNA was extracted from 10 sediment samples (Supplementary Table S1) and used for amplification of the V4-V6 region of SSU rRNA genes with archaeal-specific primers, which was subsequently sequenced on the MiSeq platform. The most abundant sequences recovered were related to microorganisms within the Bathyarchaeota, Aigarchaeota, and Thermoproteales (phylum Crenarchaeota) (Fig. 1). Members of the Thermoproteales were highly abundant in all springs at WS, but were more enriched (>90% of total sequences) at higher temperatures (WS1 and WS3 versus WS0). Thermoproteales-like sequences were not detected in any of the HL hot springs. Aigarchaeotal OTUs were recovered from every hot spring in both regions except WS1 and were the dominant phylum recovered from HL11 (>40%). In contrast to previous reports of traditional methanogenic groups inhabiting WS and HL^14, 16^, our SSU rRNA library indicated that only members of the Methanomasiliiococcales family were detected in a single hot spring (HL10).

**Figure 2:**
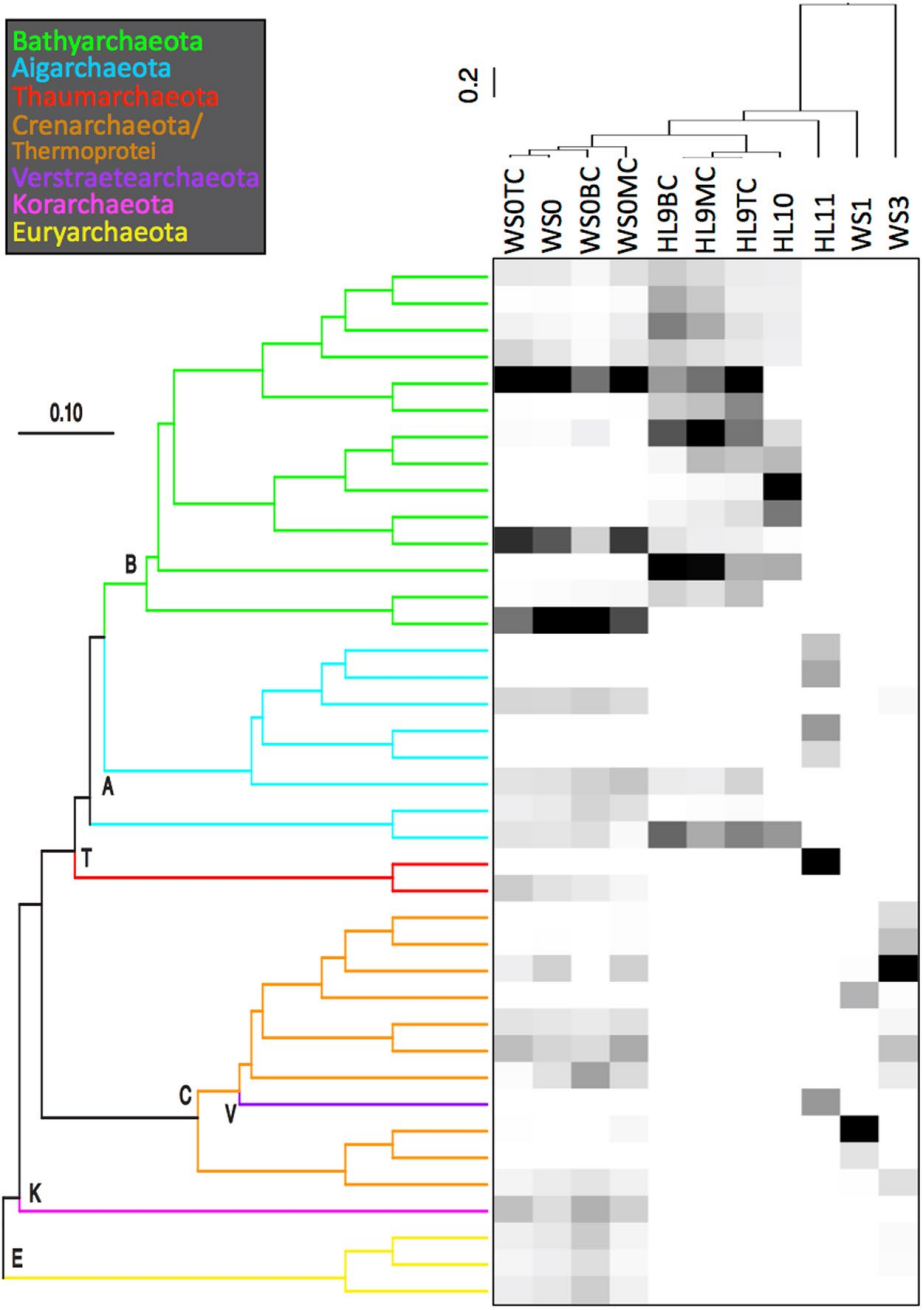
Phylogenetically resolved heat map of OTU occurrence and beta diversity across sampling sites. The relative abundance of OTUs representing >0.5% of the archaeal community is shown as a gradient from black (high abundance) to white (low abundance). A comparison of phylogenetic diversity for each sampling site is represented as a weighted UniFrac UPGMA dendogram of jackknifed phylogenetic resampling of the rarified OTU table, and demonstrates the major clustering patterns among hot springs from varying hydrothermal regions. OTU relationships are presented as a neighbor-joining tree with justified branch lengths in which branches from the same phylum are the same color corresponding to the legend. For clarity, the first letter of each phylum is placed at its respective node (B = *Bathyarchaeota*; A = *Aigarchaeota*; T = *Thaumarchaeota*; C = *Crenarchaeota*; V = *Verstraetearchaeota*; K/E = *Korarchaeota*/*Euryarchaeota*). Following the sample IDs, the designations “TC”, “MC”, and “BC” stand for “top core”, “middle core”, and “bottom core”, respectively. NB: The paraphyletic groupings of *Crenarchaeota* and *Verstraetearchaeota* are resolved in the full phylogenetic tree that includes long fragment reference sequences (Supplementary Figure S1).

High-temperature (73–77 °C) springs from WS and HL were dominated by different archaeal sequences: HL11 was characterized by sequences from the Aigarchaeota, Thaumarchaeota, and Verstraetearchaeota, while WS1 contained high amounts of different members within the Thermoproteales (phylum Crenarchaeota). Differences in the relative abundance of specific archaea between thermal regions are consistent with the marked differences in geochemistry and subsurface hydrothermal sources. Although the occurrence of Thermoproteales, Thaumarchaeota, Korarchaeota, and Euryarchaeota varied considerably across the lower-temperature hot springs sampled (i.e., <70 °C), 50–90% of recovered sequences from both thermal regions (*i.e*., WS0, HL9, and HL10) were members of the Bathyarchaeota (Figs 1 and 2). These hot springs represent a temperature range of 34–65 °C, a pH range of 6.5–8.6, diverse source gas compositions (Table 1), and distinct hydrothermal and geochemical conditions including DS content and O_2_ availability^15, 16^ (Supplementary Table S3). Despite these diverse physicochemical attributes, seven samples were dominated by bathyarchaeotal subgroups MCG-2, -6, -15^20, 21^, and -20 (Supplementary Figure S1; Fig. 1). However, closer examination reveals that distinct bathyarchaeotal phylotypes characterize WS versus HL (Fig. 2). Moreover, two subgroup-level lineages (MCG-10 and MCG-14^20, 21^) were recovered in greater abundance from the HL springs relative to WS0 (Fig. 1). Only a small fraction (<0.1%) of Bathyarchaeota-like sequences were recovered from hot springs above 70 °C (*i.e*., WS1, WS3, and HL11).

Considering the broad global and ecological distribution of members within the Bathyarchaeota^7, 20, 22, 23^, it is not surprising that different members of this phylum are distributed across distinct hot spring environments, and is supported by the potential to metabolize a broad range of substrates^6, 24^. Previous studies have concluded that many carbon sources may sustain growth of different Bathyarchaeota, including acetate^24, 25^, protocachuate^26^, glycine, urea, lipids^24^, extracellular plant-derived carbohydrates, and proteins^6, 27^. Further, Bathyarchaeota have consistently been affiliated with the degradation of complex organic matter in sedimentary environments^20, 26, 28, 29^. The hot springs sampled in this study are within topographic depressions and are surrounded by vegetated soils (Fig. 1); grasses were observed in the cores of both HL9 and WS0. Ecophysiological relationships between certain bathyarchaeotal subgroups and plant-derived complex organics^27^ have been noted, which suggests that these archaea may be metabolically linked to photosynthetically-derived organic matter. Further, previous measurements of dissolved organic carbon (DOC) at WS0 indicated concentrations up to 330 μM^30^ and in HL9 DOC was detected at concentrations as high as 450 μM at the time of sampling (Supplementary Table S3). In part, these observations may explain the high abundance of Bathyarchaeota in these specific springs.

**Figure 3:**
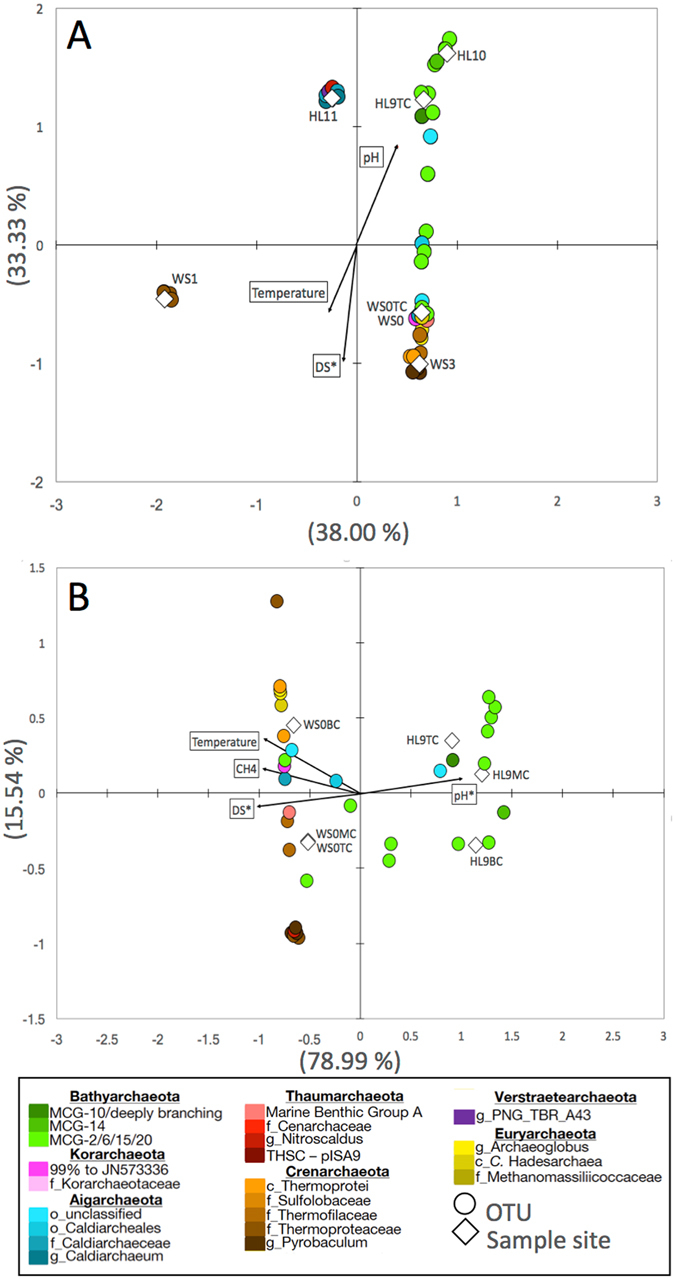
Canonical Correspondence Analyses of phylogenetic distribution and major geochemical parameters. Surface samples are compared to temperature, pH, dissolved sulfide (DS) and plotted with OTU distribution (**A**). Top, middle, and bottom sediment layers from core samples are compared to temperature, pH, DS, and porewater CH_4_ concentrations and plotted with OTU distribution (**B**). OTUs representing >0.5% sequence abundance are indicated by circles. Colors correspond to those in Fig. 1. White diamonds indicate sample sites. Black arrows indicate physicochemical parameters.

Canonical Correspondence Analysis (CCA) was used to examine the distribution of phylotypes among sites as related to major physicochemical characteristics—temperature, pH, and DS (Fig. 3). A strong division between the HL and WS geothermal regions was apparent whether only surface samples or only core samples were considered, which demonstrates their widely divergent physicochemical structure. This structure is represented clearly by contrasting directional assignments of temperature, pH, and DS along the major differential distribution pattern of OTUs from HL versus WS. Site WS1 falls outside of this pattern and contains OTUs belonging to the family Thermoproteaceae, which appear controlled by unmeasured variable(s). HL11 is surrounded by a tight cluster of OTUs related to the Aigarchaeota, Thaumarchaeota, and Verstraetearchaeota that also fits within the major physicochemical distribution pattern. Interestingly, while other phyla seem controlled by regional differences, the Aigarchaeota and Bathyarchaeota include OTUs that span the range of thermal and chemical variables. The ability of Bathyarchaeota to span such a wide range of conditions is consistent with the wide range in environmental distribution that has been repeatedly demonstrated for this phylum^7, 20, 21, 23^. However, while phylum-level occurrence spans a wide physicochemical range, OTU-level occurrence indicates that different groups within this phylum are distributed along gradients of temperature, pH, and DS. This observation underscores the importance of not generalizing the physiology or ecological preference at the phylum level, especially for a phylum as widespread and diverse as the Bathyarchaeota.

CCA was also performed on core samples to understand the distribution patterns as a function of depth into hot spring sediments. The strict delineation between HL and WS regions is again apparent, which suggests that the underlying hydrothermal and geochemical regimes are influencing microbial diversity. Detailed inspection reveals that the WS site contains a higher abundance of different archaeal phyla, whereas the sediment layers at HL are dominated by different OTUs from the Bathyarchaeota. While Bathyarchaeota is the predominant phylum at both sites, this predominance is represented by only a few OTUs in WS0 sediments versus several OTUs in HL9. CH_4_ concentrations in sediment porewaters are higher in WS0 than HL9 (Fig. 3B, Supplementary Figure S3), which is correlated with the differential regional distribution patterns. This may reflect more microbial methanogenesis in WS0 sediments, but additional data are needed to assess the proportions of geologically and biologically generated CH_4_ at both sites.

### SSU sequence recovery from reverse transcribed RNA

Total RNA was recovered from the surface sediments of HL9, and the V4-V6 region of SSU rRNA was reverse transcribed, amplified, and sequenced for comparison with the DNA-derived archaeal community (Supplementary Figure S4). The total proportion of the archaeal community captured by OTUs representing >0.1% was only 72% for the DNA survey and 97% for RNA. Shannon-Weiner diversity estimates for OTU libraries from RNA versus DNA (rarefied to equal sequence depth) were 5.6 and 7.8, respectively. This is consistent with the fact that RNA is more representative of active cells^31^, while DNA may remain after cell death^32^. Alternatively, these discrepancies might be explained by variations in extraction efficiencies of RNA versus DNA, or different RNA: DNA ratios across taxa. While some archaeal groups were underrepresented in the DNA pool (*e.g*., Thaumarchaeota, Aenigmarchaeota; supplementary text), both RNA and DNA amplicon libraries at HL9 were predominated by Bathyarchaeota (82% and 52%, respectively) belonging to subgroups MCG-2, -6, -10, -14, -15^20, 33^, and -20 (present study; Supplementary Figure S1). Consequently, results from the RNA library of HL9 support the conclusion that Bathyarchaeota are not only present, but predominate the active microbial populations.

### Occurrence of *mcrA* genes and transcripts

**Figure 4:**
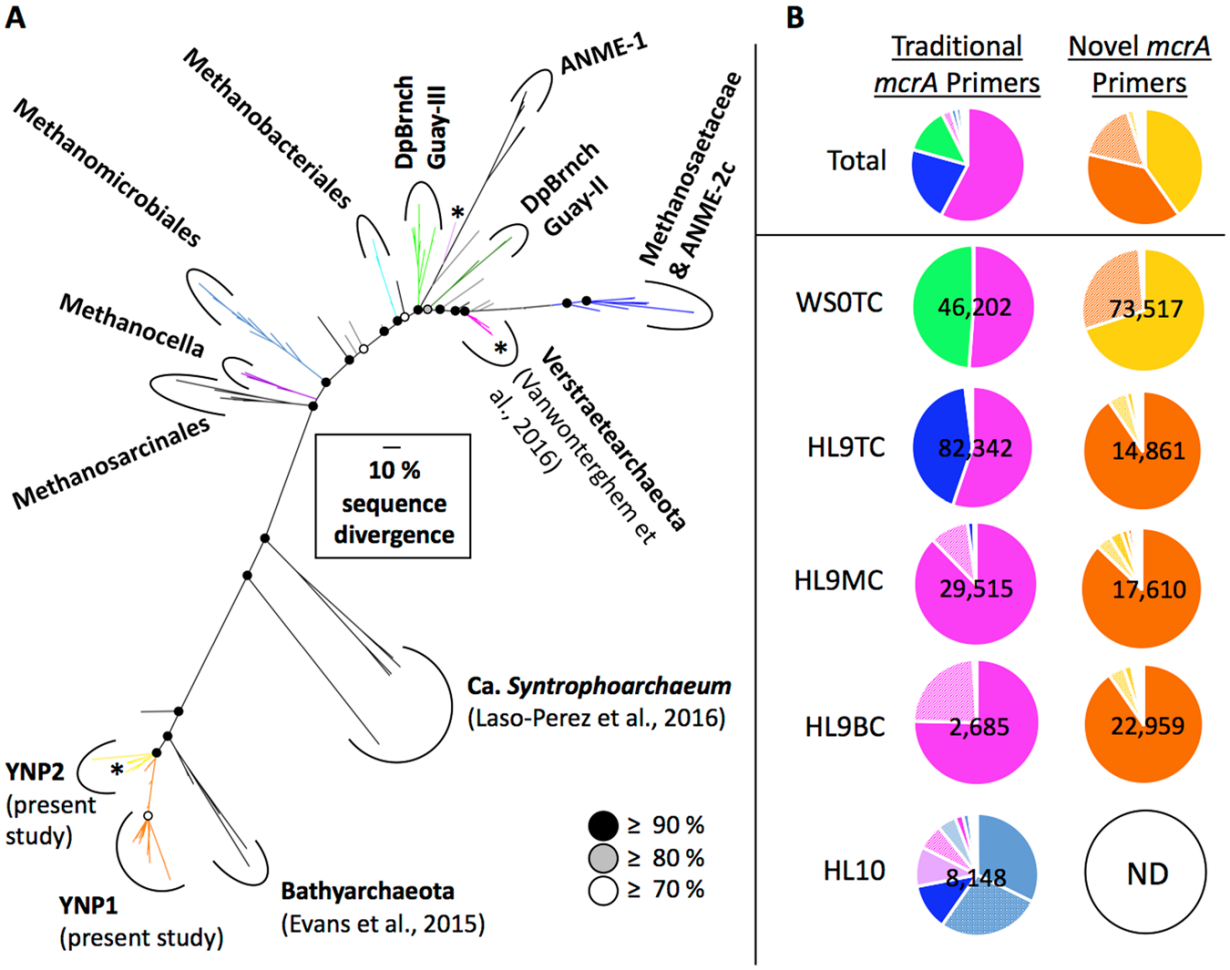
Phylogeny and occurrence of Bathyarchaeota-like (orange and yellow), verstraetearchaeotal (pink), and euryarchaeotal (other colors) McrA proteins. (**A**) Bayesian analysis of McrA phylogeny based on 150 amino acid positions. Heavy weighted branches indicate sequences recovered in the present study. Asterisks denote *mcr*A OTUs that were also recovered as RNA transcripts. Posterior probabilities are indicated by black, grey, or white circles at each node corresponding to ≥90%, ≥80%, and ≥70%, respectively. (**B**) Site specific relative abundance of *mcrA* OTUs produced by traditional and newly designed primer sets. Following the sample IDs, the designations “TC”, “MC”, and “BC” stand for “top core”, “middle core”, and “bottom core”, respectively. Total sequence number for each sample is given for each pie chart. No *mcr*A genes were amplified from HL10 with newly designed primers (ND = not determined). DpB﻿rnchG﻿﻿uay = deeply-branching Guaymas group. The scale bar ﻿corresponds to 0.1 amino acid substitutions per site.

We used previously published *mcrA* gene primers that targeted euryarchaeotal *mcrA* gene sequences^34^, and we designed new *mcrA* primers based on recently discovered bathyarchaeotal *mcrA* genes^4^ to examine the occurrence of *mcrA* genes across different geothermal springs (Fig. 4). Positive *mcrA* amplification was achieved from the top layer of WS0, the top, middle, and bottom layers of HL9, and the surface sediments of HL10. Amplified *mcrA* sequences were not recovered from HL11, WS1, and WS3, which is consistent with the lack of SSU rRNA gene sequences of known methane cycling taxa from these samples (Fig. 1). These results may also suggest that *mcrA* genes are limited to environments less than approximately 70 °C, or that lower abundance of this gene in high temperature samples precluded recovery via amplification. We recovered 169,736 and 128,949 *mcrA* sequences using traditional and newly-designed primers, respectively, which was based on comparison to our collection of currently published *mcrA* sequences (e value < 0.01). In contrast to highly divergent bathyarchaeotal *mcrA* genes, *mcrA* sequences associated with the recently proposed Verstraetearchaeota^35^ are relatively similar to those of the Euryarchaeota. This causes Verstraetearchaeotal McrA proteins to appear as a euryarchaeotal subgroup^35^ (Fig. 4), and explains why *mcrA* sequences from both phyla were amplified by the same primer set. We grouped *mcrA* OTUs at 90% nucleotide identity for sequences amplified by traditional primers, and at 97% for novel *mcrA* sequences to populate the disproportionately sparse side of the McrA tree (Fig. 4). This definition yielded 35 *mcrA* OTUs related to Euryarchaeota and/or Verstraetearchaeota and 61 *mcrA* OTUs which were basal to bathyarchaeotal sequences published by Evans *et al*. (2015).

Previous reports of euryarchaeotal *mcrA* recovery from terrestrial geothermal systems include two clones from the order Methanobacteriales from Tunisian hot springs^12^ and *mcrA* sequences related to several methanogenic genera from hot springs in Kamchatka, Russia and São Miguel Island in the Azores^36^. The latter were related to the *Methanothermobacter*, *Methanothermus*, *Methanothrix*, Methanomassiliicoccales, Methanocellales, *Methanomethylovorans*, and the uncharacterized group, MCR-2A^36, 37^. In our study, *mcrA* sequences were recovered from euryarchaeotal orders Methanocellales, Methanomicrobiales, Methanobacteriales, and Methanosarcinales. Within the Methanosarcinales, we detected an *mcrA* OTU with 92% sequence identity to that of *Methanomethylovorans hollandica* (DSM15978). This, together with our recovery of *mcrA* sequences from the Methanocellales, supports the recent conclusion that these methanogens can be found in terrestrial geothermal springs^36^. Additionally, we observed high relative abundance of *mcrA* sequences related to the uncharacterized deeply-branching Guaymas (DBG) group III, and detected group II in lower relative abundance. DBG *mcrA* clusters, which were discovered in a hydrothermal seep environment at a depth of 2000 m in the Gulf of California^38^, are functionally uncharacterized and have not been recovered previously from terrestrial systems. We did not detect sequences belonging to the MCR-2A group^36, 37^ (nucleotide identity <77%), which indicates that considerable differences exist in the ecological distribution of methanogens across globally separated terrestrial geothermal environments.

The most abundant *mcr*A OTU amplified using traditional primers was most similar to *mcrA* sequences belonging to the recently proposed Verstraetearchaeota^35^; this OTU was observed in every sample where *mcrA* sequences were successfully amplified. However, no SSU rRNA gene sequences affiliated with the Verstraetearchaeota were detected in these hot springs (*i.e*., WS0 and HL9). In contrast, the SSU RNA gene library from HL11 contained a large proportion of SSU rRNA gene sequences belonging to the Verstraetearchaeota, yet no verstraetearchaeotal *mcrA* sequences were recovered. These results suggest that (i) additional studies are necessary to determine whether some members of Verstraetearchaeota encode *mcrA* gene variants that are phylogenetically distinct from those recently described^35^, (ii) not all members of the Verstraetearchaeota harbor *mcrA* genes, and/or (iii) most SSU rRNA gene primers currently used in next generation taxonomic diversity studies severely underestimate archaeal diversity^39^. Previously identified Verstraetearchaeota were inferred to be methylotrophic methanogens capable of utilizing methanol, methanethiol, and methylated amines^35^, and we hypothesize these could be important metabolic modes in YNP hot springs as well.

In surface sediments of HL9, 43% of *mcrA* sequences belonged to an OTU most similar to the Methanosaeta, one of two known groups capable of acetoclastic methanogenesis^40^. The next most abundant OTU, which contained 49% of the *mcrA* sequences from WS0 surface sediments was related to the DBG group III, a functionally uncharacterized *mcrA* clade originally discovered in the hydrothermal sediments of Guaymas Basin^38, 41^. Another Verstraetearchaeota-like sequence was recovered in high abundance in the HL sediments, and the most abundant *mcrA* OTU at the cooler hot spring (HL10) grouped within the Methanomicrobiales, which harbors hydrogenotrophic, formate-utilizing methanogens^42^. Despite the cultivation of *Methanothermobacter thermautotrophicus* from WS0 sediments^14^, we observed low relative abundance of *mcrA* sequences belonging to the Methanobacteriales order. Instead, traditional *mcrA* primers suggest that WS0 is dominated almost entirely by members of the Verstraetearchaeota and the DBG III group^38^. We recently cultivated *M. thermautotrophicus* from WS0 using the same sediment samples (unpublished data), which indicates that this organism may be easily captured by classical cultivation methods for hydrogenotrophic methanogens, but does not represent the ecologically predominant population.

Using newly developed primers targeting recently reported bathyarchaeotal *mcrA* genes^4^, we identified two novel, deeply-branching McrA clades (YNP1 and YNP2, Fig. 4; Supplementary Figure S2). A single mcrA OTU from the novel YNP1 clade was dominant at all depths of the HL9 sediment core, whereas a single OTU from the YNP2 clade was dominant in the surface sediments of WS0. These results are consistent with the major geochemical differences between HL and WS (e.g., pH and sulfide content) that likely result in the enrichment of distinct CH_4_ cycling archaea. However, lower abundance of YNP1 and YNP2 mcrA sequences was observed in both regions, precluding regional isolation of these novel mcrA clades. Considering the closest phylogenetic relatives to mcrA OTUs recovered from the present study, it appears that all modes of methanogenesis (i.e., methylotrophic, hydrogenotrophic, and acetoclastic) may be possible by multiple archaeal phyla in YNP hot spring sediments.﻿.

Notably, only one of three newly designed *mcr*A primer sets was successful in amplification (Supplementary Table S2), and this primer set only targeted half of the recently reported *mcrA* genes. Hence, it is probable that more than three major McrA clades related to Bathyarchaeota (Fig. 4) have yet to be discovered. Further, while *mcrA* genes with the YNP1 and YNP2 clades were amplified by primers designed from sequences associated with bathyarchaeotal genomic assemblies^4^, the amino acid identities of translated amplicons to the bathyarchaeotal McrAs are only 57% and 68%, respectively. This indicates that YNP1 and YNP2 McrA clades may not belong to the Bathyarchaeota. Illustrating this point, McrAs that are established as belonging to distinct phyla, (*e.g*., sequences from Verstraetearchaeota V4^5^ and Methanomasiliicoccales (phylum Euryarchaeota)), share an amino acid identity of 68%. Thus, attributing YNP1 and YNP2 *mcrA* sequences to the Bathyarchaeota is not prudent until further investigation can test their taxonomic relationship. Moreover, given the relatively large phylogenetic distance between McrA clades, as well as the recently elucidated butane-processing capacity of *C*. Syntrophoarchaeum^8^, physiological function cannot be assumed based on phylogenetic position alone.

Lastly, three of the 96 total *mcr*A OTUs were also recovered in the reverse-transcribed RNA library from HL9. A single *mcrA* transcript grouped within the novel YNP2 clade, while the most abundant *mcrA* transcript from traditional primers was related to the Verstraetearchaeota (asterisks, Fig. 4). Transcripts were also recovered for another OTU too distantly related to the anaerobic methanotroph group 1 (ANME-1) for functional inference of methanotrophy, underscoring the need for further studies of methane cycling diversity in hot springs. The Verstraetearchaeota-like transcripts were represented by the most abundant OTU from the DNA library while the deeply-branching ANME-1 and YNP2 transcripts were recovered in very low abundance in the DNA library. These data indicate that novel and deeply-rooted *mcr*A sequences are present and actively expressed in hot spring sediments from distinct geothermal regions of YNP. Future investigations of physicochemical influences, metagenome assemblies, and carbon substrate utilization are needed to determine the capacity for methane cycling in novel archaea from high-temperature environments.

## Conclusion

We investigated physicochemically diverse thermal sites in YNP and observed an abundance of archaeal SSU rRNA gene sequences related to the Bathyarchaeota, Aigarchaeota, Crenarchaeota, Verstraetearchaeota, and Thaumarchaeota. Representatives of the Bathyarchaeota were highly abundant in both DNA and RNA libraries, indicating that members of this lineage are predominant and metabolically active in at least three geochemically and hydrothermally distinct hot springs. *mcr*A gene sequences from the Verstraetearchaeota and Euryarchaeota, and two novel clades basal to the Bathyarchaeota^4^, were observed across five distinct hot spring sediment samples using PCR primers designed to target previously unknown *mcrA* genes as well as those amplified using well established primers. In addition, *mcr*A transcripts from diverse archaeal phyla were amplified from cDNA and for the first time demonstrate that novel, putative methane cycling genes are expressed in the environment. These data corroborate the hypotheses that recently discovered, widely distributed lineages^4, 23, 35^ might have been overlooked for their role in environmental methane cycling. The presented data greatly expand the diversity of a key biomarker for methanogenesis, anaerobic methane oxidation, and higher alkane utilization, and confirm that at least two archaeal phyla likely contribute to global methane fluxes.

## Methods

### Site selection and sample collection

Washburn Hot Springs (WS) and Heart Lake Geyser Basin (HL) contain thermal features that span a wide range of physicochemical variables^15^, including temperatures from 34 °C –91 °C, pH values from 5.8–8.6, and dissolved sulfide concentrations from detection (HL) to 231 µM (WS). While the geochemical parameters that characterize WS and HL are widely divergent (Table 1; Supplementary Table S3), these geothermal regions were selected because they are, to our knowledge, two of three sites in YNP where methanogenesis has been suggested^14, 15^.

Surface sediments (to 1 cm) were collected from two hot springs at WS (WS1 and WS3), and two springs at HL (HL10 and HL11) in 50 ml falcon tubes. Sediments from a third hot spring from each region that exhibited similar temperature near 60 °C (WS0 and HL9) were sampled by PVC push coring. Three sections from each core (top, middle, and bottom) were subsampled for DNA and porewater gas analysis (Supplementary Figure S3). For DNA analyses, 50 ml of sediment was immediately placed on dry ice and subsequently stored at −80 °C in the lab until extraction, and for RNA, sediments were preserved in RNAlater solution (1:1) at the time of sampling and stored at 4 °C until extraction. Geothermal source gases were sampled from hot spring bubblers in quadruplicate at WS0, WS1, HL9 and, due to weather and time constraints, only once at HL10. This sampling method was not possible at WS3 or HL11, because the water depth was not sufficient to immerse serum bottles. Gas samples of 240 ml were stored in preevacuated 160 ml serum vials with 1 ml 2.5 M sodium azide and stored upside down until analysis. Sediments from core sections were sampled for porewater CH_4_ concentrations by injecting 5 ml sediment into a 30 ml serum vial containing 2 ml of 1 M NaOH, which were then sealed with blue butyl stoppers, shaken vigorously, frozen on dry ice, and stored upside down at −20 °C until laboratory analysis^43^. In an effort to make all analyses (geochemical and molecular biological) on identical portions of sediment, core sediments were slurried in 5-cm sections for a total of 10 sediment samples per core (Supplementary Table 1). Prior to sediment coring, a thermal profile was retrieved adjacent to the coring location by attaching the thermocouple wire ends from a Fluke model 52–2 (60 Hz) dual input digital thermometer to the end of a wooden rod marked with 5-cm hashes, which was then pushed into the sediments stopping for 1 min at each 5-cm interval to record temperatures.

### Gas collection and analyses

A Varian micro gas chromatograph (CP2900) equipped with molecular sieve and chemical separation columns (thermal conductivity detection) was used to determine concentrations of H_2_, CH_4_, carbon monoxide, and CO_2_ in gas samples obtained from geothermal source pools and only CH_4_ for porewater gases.

### DNA and RNA extraction and amplification

DNA was extracted from approximately 10 g of sediment using the PowerMax Soil DNA kit from MoBio (Carlsbad, California, USA). Samples from which DNA yield was low were re-extracted with the FastDNA Spin Kit for Soil from MP Biomedicals (Santa Ana, California, USA). The SSU rRNA gene was amplified using a previously published PCR protocol^15^ with the Bullseye Taq DNA polymerase from MidSci (St. Louis, Missouri, USA) and archaeal primers 751 F and 1204R^44^ with Illumina MiSeq adapter sequences on the 5’ ends.

Euryarchaeotal *mcrA* genes were targeted using previously tested primers developed from a database of 5200 *mcrA* sequences and amplified by touchdown PCR^34^. In the present study, these primers were found to amplify newly discovered verstraetearchaeotal *mcrA* genes as well as those from the Euryarchaeota, and thus have a broader coverage than previously reported^34^. For detection of novel *mcrA* genes related to the Bathyarchaeota we manually designed primers in ARB v6.0.6^46^ based on the 19 currently available sequences from the supplementary information of recent work by Evans *et al*. (2015; Supplementary Text). We targeted three distinct phylogenetic subgroups related to bathyarchaeotal *mcrA* genes (Supplementary Table 2; Supplementary Text).

Consistent with reports from the literature that successful *mcrA* amplification often necessitates cumulatively high cycle numbers following touchdown PCR (e.g., 35–40 cycles^46^, 40 cycles^38^, 45 cycles^47^), we used Bullseye Taq DNA polymerase in amplification of Bathy-*mcrA* group 2 genes with the following touchdown PCR protocol: initial denaturation and enzymatic activation at 95 °C for 15 min, followed by 2 cycles of 20 sec at 95 °C, 30 sec at 52 °C, and 45 sec at 72 °C, 2 cycles of 20 sec at 95 °C, 30 sec at 50 °C, and 45 sec at 72 °C, 2 cycles of 20 sec at 95 °C, 30 sec at 48 °C, and 45 sec at 72 °C, 2 cycles of 20 sec at 95 °C, 30 sec at 46 °C, and 45 sec at 72 °C, 32 cycles of 20 sec at 95 °C, 30 sec at 45 °C, and 45 sec at 72 °C, and a final extension of 5 min at 72 °C. The only successful amplification was achieved with the group 2 primers (Bathy_mcrA_2/3 F and Bathy_mcrA_2 R; Supplementary Table 2). Due to the high sequence divergence between McrAs from *Ca*. Syntrophoarchaeum (Laso-Perez *et al*., 2016) and Bathyarchaeota (Evans *et al*., 2015), exhibiting a closest possible amino acid identity of 48%, the Syntrophoarchaea were not targeted by our primer sets. PCR products of the correct length (ca. 450 bp for SSU rRNA genes and ca. 500 bp for *mcrA* genes) were excised from a 1% tris-acetate-ethylenediaminetetraacetic acid gel and purified using the Wizard Gel/PCR cleanup kit from Promega (Madison, WI, USA) and were stored at −80 °C prior to sequencing. In some cases, non-specific primer binding yielded an additional band at 750 bp; when sequenced, this product was identified as a member of the tRNA-guanine-transglycosylase (TGT) complex. Interestingly, this particular TGT protein was related to previously sequenced bathyarchaeotal TGTs, which suggests proximal association between TGT and *mcrA* loci.

Prior to RNA extraction, two parts of RNALater-preserved sediment solution were diluted in one part 1X PBS and centrifuged for 5 minutes at 6,000xG. The supernatant was discarded and total nucleic acids were isolated per previously published methods^48^. From the total RNA/DNA pool, RNA was subsequently purified starting from Step 6 in the manufacturer’s protocol of the FastRNA Pro Soil Direct kit from MP Biomedicals (Santa Ana, CA, USA). To eliminate DNA contamination samples were then treated with DNase using the Turbo DNA-free kit from Thermo Fisher Scientific with the suggested protocol for “rigorous” DNase treatment. This RNA isolation method was attempted on multiple replicates of every sediment sample but only yielded quantifiable RNA from the surface sediments of HL10 and the top of the HL9 sediment core. On RNA from the top of HL9, the absence of DNA was confirmed by performing PCR amplification of SSU rRNA genes on an aliquot of RNA using the previously mentioned protocol and positive controls with DNA. The remaining RNA was reverse-transcribed by first strand synthesis with archaeal SSU rRNA primers mentioned above and the SuperScript III kit from Thermo Fisher Scientific according to the manufacturer’s protocol, with the exception that RNasin (Promega, Madison, WI, USA) was used instead of RNaseOUT but at the same suggested concentration. PCR amplification of reverse-transcribed cDNA was then performed as it was for DNA extractions (above) with the exception that MgSO_4_ was added to a final concentration of 1.5 mM to offset the removal of Mg^2+^ ions by EDTA during DNase treatment.

For detection of *mcr*A RNA transcripts, we performed primer-targeted first strand synthesis using the SuperScript III reverse transcriptase kit mentioned above. For euryarchaeotal *mcr*A transcripts we used the manufacturer’s protocol for “gene specific reverse transcription” together with the previously mentioned euryarchaeotal *mcr*A primers. For bathyarchaeotal *mcr*A transcripts, a “touchdown” first strand synthesis with bathyarchaeotal primers (Supplementary Table 2) and SuperScript III reverse transcriptase was used in which we followed the gene specific protocol but, instead of incubating at 55 °C for 60 min, we incubated at 55 °C for 20 min, 50 °C for 20 min, and 45 °C for 20 min, followed by a 15 min enzyme inactivation at 70 °C. PCR amplification of *mcr*A cDNA was then performed as previously described for *mcr*A DNA.

### Sequencing and analysis

Purified SSU rRNA and *mcrA* gene amplicons (450 bp to 500 bp) were sequenced on an Illumina MiSeq platform (San Diego, CA, USA) following preparation according to the “16S Metagenomic Sequencing” protocol for 300-read paired end sequencing. Following original PCR, amplicons with MiSeq adapter sequences were purified with Ampure XP beads and then index PCR was performed to ensure identification of samples post-pooling. Indexed amplicons were purified with an additional bead cleanup and concentrations were determined for DNA aliquots stained with PicoGreen (Quant-IT, Invitrogen, Carlsbad, CA, USA). Sample concentrations were normalized and all samples were pooled and mixed with the PhiX control library at 10% of the sample DNA concentration. The mixed sample-PhiX DNA was then loaded on the Illumina MiSeq and sequenced.

Forward and reverse reads for SSU rRNA genes were assembled using QIIME^49^ (MacQIIME v1.9.1) with the split_libraries_fastq.py command based on previously tested default parameters^50^, which resulted in 1,942,987 QC-passed reads. The lowest sequence count for any sample was 52,130 for WS0_TC. OTUs were picked using the open reference picking method in QIIME^49, 51^ and initial taxonomic assignments were made with UCLUST^51^ and the Greengenes reference database (gg_13_5)^52^. This produced an OTU table without singletons with a total sequence count of 1,900,511. Next, sequences that did not align via PyNAST^53^ methods were thrown out, resulting in a total of 604,842 sequences with a smallest sample size of 9,042 for WS0BC. OTUs generated from sequenced extraction blanks and negative PCR controls were pooled and used to filter out contamination-based OTUs from the total dataset, decreasing sequence counts to 582,343 and 8,087, respectively. Finally, OTUs were further filtered for chimeric sequences with UCHIME^54^ in Mothur^55^, which reduced the total archaeal sequence count from 582,342 to 543,797 sequences and resulted in a minimum of 8,080 sequences for WS0_BC. This quality-filtered dataset was then rarified to 8,080 sequences per sample by random sampling with the single_rarefaction.py command in QIIME^49^. Rarefaction curves are provided to demonstrate sampling depth, OTU observations, and Shannon-Weiner^56^ diversity estimates for the total, non-rarified OTU table as well as the rarified OTU table (Supplementary Figure S6). All downstream analyses of relative abundance, phylogeny, and beta diversity were performed on the OTU dataset rarified to 8,080 sequences per sample. PCoA plots of weighted UniFrac^57^ metrics were created in QIIME^49^ with the beta_diversity.py and make_2D_plots.py commands to demonstrate that similar patterns of beta diversity are revealed by each OTU cutoff value used in the present study. Rarefaction curves were calculated and display the number of new OTUs observed per sequence for the unrarified and rarefied datasets, and an additional rarefaction curve is presented to demonstrate Shannon-Weiner diversity estimates per sequence for the rarified dataset (Supplementary Figure S6). Shannon-Weiner diversity estimates were calculated in QIIME^49^ with the alpha_diversity.py function according to default parameters described at scikit-bio.org. Diversity estimates were calculated the same way for DNA and RNA generated libraries from HL9TC after being normalized to a maximum common sequence count of 3,609.

For detailed taxonomic assignments and phylogenetic relationships, reference sequences were selected based on closest BLAST identities to recovered OTUs as well as a priori knowledge of relevant type strains or well-characterized long-fragment SSU genes. OTUs representing 0.5% or more of all sequences were aligned with reference sequences using the SINA online alignment platform and the v1.2.11 database^58^ with default parameters. SINA-based alignments were manually inspected and edited in ARB v6.0.6^46^. A maximum-likelihood phylogenetic tree of near full length SSU reference sequences (>1100 bp) representing as many recovered groups as possible was generated in ARB^45^ with the rapid bootstrap RAxML algorithm with 1000 iterations. GTRGAMMA was selected as the rate distribution model because it resulted in strong bootstrap support of previously understood phylum level lineage relationships. OTUs from this study were added to the maximum likelihood reference tree with the ARB parsimony methods^45^. The colored dendogram in Fig. 2 was generated only for OTUs > 0.5% sequence recovery (no reference sequences) by ARB neighbor-joining methods^45^ with default parameters followed by branch transforming. UniFrac^57^ jackknifed beta diversity for all samples was performed for the rarified OTU table (8,080 sequence depth) and is displayed as a weighted UPGMA tree. Weighted UniFrac^57^ sample diversity was calculated with the beta_diversity_through_plots.py function in QIIME^49^ on four different OTU tables representing the different cutoff values presented in this work (Supplementary Figure S5). CCA plots were created in Microsoft Excel v15.33 (Redmond, WA, USA) with the XLSTAT add-in v19.03 (New York, NY, USA) and default parameters for the rarified OTU table and associated physicochemical parameters for each sample location. Each CCA was run at 1000 permutations and a significance level of 5%.

Forward and reverse *mcrA* sequences were assembled and quality-filtered in QIIME^49^ using the split_libraries_fastq.py command with default parameters as described above. Next, sequences that failed a BLAST v2.2.22 (National Center for Biotechnology Information, USA) search against a reference collection of euryarchaeotal, verstraetearchaeotal, and bathyarchaeotal sequences at a cutoff e value of 1e-10 were discarded. For the reference collection, we used all available *mcrA* sequences from Bathyarchaeota, Verstraetearchaeota, Ca. Syntrophoarchaeum, and two to three representatives from each order of known methanogens within the Euryarchaeota as well as sequences belonging to uncultured anaerobic methanotrophs. We selected a liberal e value to capture as much novel *mcrA* diversity as possible, but manually inspected alignments in ARB^45^ and discarded non-*mcrA* sequences following OTU picking. Euryarchaeotal *mcr*A OTUs were picked with UCLUST^51^ at a sequence similarity of 90%, and bathyarchaeotal mcrA OTUs were picked at a sequence similarity of 97% to add more phylogenetic information to the to the comparatively sparse bathyarchaeotal side of the phylogenetic tree. All *mcrA* gene sequences were translated to amino acid sequences with ORF-Predictor (http://bioinformatics.ysu.edu/tools/) and aligned with MAFFT^59^ to our reference collection, and chimeric assemblies were identified visually in ARB^45^ and discarded. Initial neighbor-joining trees were constructed in ARB to identify sequences with no close relatives, and a web-based BLAST search (National Center for Biotechnology Information, USA) was performed to retrieve closer relatives where possible. Bayesian phylogenetic analyses of amino acid McrA sequences was performed using MrBayes v3.2^60^. To guarantee similar lengths of protein sequences, a termini filter was applied, and all characters were considered during tree reconstruction. Model-jumping between fixed rate models was allowed during protein tree calculations. The WAG model – a protein evolution model that uses unequal but fixed stationary state frequencies and substitution rates – was found to best describe the McrA dataset. A total of 41 million trees were calculated. At the end of the analyses, the standard deviations of split frequencies between the two chains had reached a value of 0.035. Parameter values and trees were summarized using a burn-in of 25%. The final potential scale reduction factor value was 1.00. Taxonomic associations of sequenced *mcrA* genes were determined by neighboring reference sequences in the Bayesian tree.

### Data availability

The sequence datasets generated from the current study are available from the NCBI repository, under BioProject ID PRJNA393290 for SSU rRNA amplicons from DNA and RNA, and BioProject ID PRJNA393291 for *mcrA* amplicons from DNA and RNA.

## Electronic supplementary material

Supplementary Information
