# Supplementary material for: Occurrence and expression of novel methyl-coenzyme M reductase gene (mcrA) variants in hot spring sediments
Source: Sci Rep. 2017 Aug 3;7:7252. doi: 10.1038/s41598-017-07354-x (PMC5543129; doi:10.1038/s41598-017-07354-x)
Supplement: Supplementary file 1 — Supplementary Information [file 41598_2017_7354_MOESM1_ESM.doc]

**Supplementary Information**

**Title:** Occurrence and expression of novel methyl-coenzyme M reductase gene (*mcrA*) variants in hot spring sediments

**Authors**: Luke J. McKay^1,2*^, Roland Hatzenpichler^1,3^, William P. Inskeep^2^, and Matthew W. Fields^1,4*^

**Affiliations**: ^1^Center for Biofilm Engineering, Montana State University, Bozeman, MT 59717

^2^Department of Land Resources and Environmental Sciences, Montana State University, Bozeman, MT 59717

^3^Department of Chemistry and Biochemistry, Montana State University, Bozeman, MT 59717

^4^Department of Microbiology and Immunology, Montana State University, Bozeman, MT 59717

Key words: methane cycling, Bathyarchaeota, Verstraetearchaeota, geothermal

Corresponding authors:

L.J. McKay, PhD

313 Barnard Hall

Center for Biofilm Engineering

Montana State University

Bozeman, MT 59717

luke.mckay@montana.edu

M.W. Fields, Professor 366 Barnard Hall

Center for Biofilm Engineering

Montana State University

Bozeman, MT 59717

matthew.fields@biofilm.montana.edu

**NB:**

The springs at HL were previously referred to by their temperatures at the time of sampling for the investigation by De León et al.: 44°C, 63°C, and 75°C (2013), which correspond to HL10, HL9, and HL11 in this study. Notably, at 34°C HL10 was 10°C cooler when sampled for the present study than the previous study by De León et al. (2013). At WS, the spring designated “WS0” in this investigation is the same spring sampled by Inskeep et al., which was called “WS_18” (2013). To our knowledge the microbial ecosystem of the other two springs, WS1 and WS3, has not been previously investigated.

**Results & Discussion**

Push cores retrieved from WS0 and HL9 and cut into three sections (Supplementary Table 1) revealed remarkable archaeal community similarity downcore. UniFrac metrics of β diversity, including weighted principal coordinate analyses and jackknifed resampling of overall phylogenetic diversity within samples, confirmed that separate sections of the sediment cores more resemble environmental replicates than distinct samples (Figure 2; Supplementary Figure S4). At HL9, even when all OTUs comprised of n > 1 sequences are considered, every layer of the HL9 core as well as the sediments from HL10 cluster very tightly (Supplementary Figure S4); this indicates that the background, low-abundance microbial community is consistent throughout the cooler Heart Lake hot springs. At Washburn, the top layer of the WS0 core is tightly clustered with a replicate non-core sample from the surface sediments of WS0 while the middle and deep sediment layers do not cluster closely in principal coordinate space. In other words, differential communities exist among separate layers of the WS0 core when the larger, background community is considered. All sediment communities from WS0, HL9, and HL10 are more tightly clustered with one another than they are to WS1, WS3, and HL11 (Supplementary Figure S4), presumably resulting from marked differences in temperature. On the other hand, jackknifed phylogenetic resampling of the high-abundance community (n > 0.5%) indicates that the HL9 and HL10 samples are more similar to HL11 than they are to any Washburn samples (Figure 2). This is surprising given the predominance of Bathyarchaeota in both regions but not in HL11, but can be explained by differences between the dominant bathyarchaeotal OTUs at WS versus HL as well intraregional similarities in non-bathyarchaeotal groups (e.g., Thermoprotei are primarily associated with WS samples).

The bathyarchaeotal majority in the two push core samples, WS0 and HL9, extends from the surface layer to the deepest layer sampled, which may reflect a wide range of electron acceptor possibilities for bathyarchaeotal metabolisms. However, the “surface layer” of the push cores represents a range of 0 – 5 cm, and thus the predominance of Bathyarchaeota may be more representative of the sediments just below the surface than within the actual phototrophic mat community at the sediment-water interface. Indeed, while the archaeal community is consistent downcore, the bacterial community at the surface was representative of a phototrophic mat community (Roseiflexus, Gloeobacter, etc; unpublished data). These deeper layers may also serve as buffers to sources of oxidative stress{Lazar:2016fe} and/or thermal activity where temperatures are slightly less than values from surficial fluids (Supplementary Figure S3). The UniFrac β diversity metrics (Figure 2; Supplementary Figure S4) also indicate a clear distinction between thermal regions in which the phylogenetic diversity is more similar among all HL springs than between any HL and WS hot spring. However, the same is not true for WS, where WS0 is more similar to HL springs than to either WS1 or WS3, likely due to the predominance of the Bathyarchaeota.

In addition to sampling the top of the sediment core from WS0, we sampled surface sediments directly from the hot spring (Supplementary Table S1) to assess how different sampling techniques of the same site capture phylogenetic diversity. While the rarefaction curve for WS0TC is much steeper than for WS0 (Supplementary Figure S6), CCA (Figure 3), PCoA (Supplementary Figure S5), and UPGMA (Figure 2) analyses each separately demonstrate a tight clustering of these replicates. In addition, tightly clustered groups for top, middle, and bottom layers of sediment cores from the same figures also indicate the reproducibility of SSU rRNA recovery. Since every analysis of beta diversity demonstrates that all other samples are more distantly related than either WS0 and WS0TC or distinct layers of the same sediment core, we maintain that our sampling design was appropriate for distinguishing between phylogenetic signals from different sample sites.

*Differential recovery of SSU rRNA groups in DNA versus RNA.*

Several lineages represented > 0.1% of sequences in the RNA pool but not the DNA pool, including sequences related to the Aenigmarchaeota (formerly Deep Sea Euryarchaeotal Group), *Nitrosocaldus* spp. (phylum *Thaumarchaeota*), unclassified Thermoprotei (Crenarchaeota), and *Archaeoglobus* spp. (Euryarchaeota) (Supplementary Figure S4). The largest of these groups were comprised of sequences within the Thaumarchaeota and Aenigmarchaeota, which represented 14.9 % and 10.7 %, respectively, of the total RNA library from the surface sediments of HL9. This indicates that these groups are more active in the community than is suggested by relative abundance SSU genes from DNA.

*Primer design for detection of novel* mcrA *genes*

We restricted the forward and reverse loci to the close vicinity of traditional primer sites to yield a similar amplicon region for downstream alignments, and we ensured that each primer set had similar melting temperatures (Supplementary Table S4). Our primers range in length between 18-20 bp and each has an even distribution of G/C bases while none have > 3 G/C bases in the 5 bp termini. No consensus primer sequence was possible for all 19 bathyarchaeotal sequences, which resulted in three primer sets targeted at distinct bathyarchaeotal subgroups. For group 1, Bathy_mcrA_1F and Bathy_mcrA_1R primers were designed to target *mcrA* gene sequences KT387817, KT387819, KT387808, and KT387809. A single forward primer, Bathy_mcrA_2-3F, was designed to target bathyarchaeotal groups 2 and 3, which consist of KT387813, KT387818, KT387814, KT387816, KT387812 (group 2), and KT387806 and KT387810 (group 3). The group 2 reverse primer, Bathy_mcrA_2R, is a perfect match to KT387813 and KT387814 and the group 3 reverse primer, Bathy_mcrA_3R, matches KT387806 and KT387810.

**Supplementary Table S1 | Sediment depths sampled at each hot spring site.** Sediments were sampled for DNA extractions from three hot springs at Washburn (WS) and three at Heart Lake (HL). At WS0 and HL9 a push core was retrieved and sectioned in three layers. The depth range of all sediment samples from every site is listed in cm. RNA was extracted from the top layer (0 – 5 cm) of HL9.


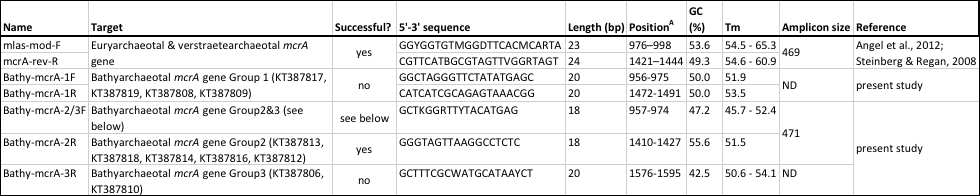


**Supplementary Table S2 | Information for published and newly designed *mcrA* primers.** Target organisms, amplification success, sequence, length, position, % GC, melting temperature (Tm), amplicon size, and reference for each primer set attempted is provided. Accession numbers of previously published genes that were targeted by each new primer set are listed, as well as which primer sets were successful. At the time of primer design, the only available sequences of *mcrA* genes belonging to *Bathyarchaeota* were from the publication by Evans et al. (2015).

^A^nucleotide position was based on the *mcrA* gene sequence of *M. thermautotrophicus* (GenBank accession U10036 following Steinburg & Regan, 2009 and Angel et al., 2012)


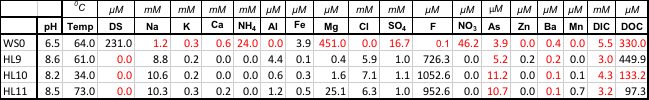


**Supplementary Table S3 | Physicochemical parameters for WS0, HL9, HL10, and HL11.** DS = dissolved sulfide, DIC = dissolved inorganic carbon, DOC = dissolved organic carbon. Values in black were determined at the time of sediment sampling for DNA. Values in red are added from historical data (from De Leon et al., 2013, Inskeep et al., 2013, or Jennings et al., 2017).

**Supplementary Figure S1 | Maximum likelihood phylogenetic relationships of OTUs based on 16S rRNA genes.** A RAxML tree was constructed in ARB with a GTRGAMMA rate distribution model and bootstrapped at 1000 iterations. The base tree consisted only of reference sequences with > 1100bp, and OTUs from the present study (ca. 500bp) representing > 0.5 % sequence abundance were added with ARB parsimony methods. Phylum-level lineages are indicated in bold to the right of phylogenetic groups. MCG-subgroups for the *Bathyarchaeota* are named according to investigations by Kubo et al. (2012) and Lazar et al. (2015), except for MCG-20, which was identified in the present study.

**Supplementary Figure S2 | Neighbor-joining clustered dendogram of *mcrA* gene sequences.** A neighbor-joining algorithm was used to cluster *mcrA* gene sequences for comparison to other *mcrA* amplicon studies, which commonly publish neighbor-joining trees. A Jukes-Cantor correction was applied and bootstrap values were calculated after 1000 tree iterations. Two newly identified bathyarchaeotal subgroups from YNP are identified as YNP1 and YNP2.

**Supplementary Figure S3 | Sediment core sample sites and adjacent profiles of subsurface temperature and methane concentration.** Photos of WS0 and HL9 hot springs indicate precise locations of sediment coring (yellow X). Thermal profiles were measured adjacent to each coring site at increments of ca. 5 cm down to 50 cm (WS0) and 30 cm (HL9). Porewater methane concentrations were measured in core sediments and demonstrate subsurface peaks in methane at both sites. Grey bars indicate sediment layers that were sampled for DNA analyses.

**Supplementary Figure S4 | SSU rRNA genes versus SSU rRNA transcripts at hot spring HL9.** (A) Relative abundance of archaeal community based on SSU rRNA *gene* recovery in HL9 surface sediments for groups that were also recovered from RNA. (B) Relative abundance of the archaeal community based on SSU rRNA *transcript* recovery in HL9 surface sediments. Group colors are consistent with Figures 1 and 2, and taxa that were recovered from RNA but not > 0.1 % for DNA (as shown in Figure 1) are in black and white patterns. Archaeal groups observed at low relative abundance are labeled on the pie charts for ease of viewing. MCG = Miscellaneous Crenarchaeotal Group (see Kubo and Lloyd et al., 2012; Lazar et al., 2015), DSEG = Deep Sea Euryarchaeotal Group (Takai et al., 2001), TMEG = Terrestrial Miscellaneous Euryarchaeotal Group (Sørensen et al., 2004).

**Supplementary Figure S5 | Principal coordinates analysis of weighted UniFrac beta diversity for different OTU cutoff values.** UniFrac weighted metric of phylogenetic diversity between hot spring sediment samples is plotted in principal coordinates space for the quality-filtered OTU table (A), the rarefied OTU table to 8,080 sequence depth (B), the rarified OTU table filtered at a > 0.1 % cutoff consistent with Figure 1 (C), and the rarified OTU table filtered at a > 0.5 % sequence cutoff consistent with Figure 2 (D). Axis labels indicate the percent variation explained by each axis. TC, MC, and BC stand for “top core”, “middle core”, and “bottom core”, respectively.

**Supplementary Figure S6 | Rarefaction curves of SSU rRNA reads.** Distinct OTUs are plotted against total sequence recovery per sample for the total quality-filtered sequence library (A), the rarefied sequence library at 8,080 sequences (B), and Shannon-Weiner diversity estimates are plotted per recovered sequence from the rarified dataset (C). Colored lines correspond to separate samples and overlapping lines are indicated by black arrows and text.
